# Supplementary material for: Immunomodulatory Activity of Punicalagin, Punicalin, and Ellagic Acid Differs from the Effect of Pomegranate Peel Extract
Source: Molecules. 2022 Nov 15;27(22):7871. doi: 10.3390/molecules27227871 (PMC9695876; doi:10.3390/molecules27227871)
Supplement: Supplementary file 1 [file molecules-27-07871-s001.zip › molecules-2004695-SM.pdf]

Supplementary Figure S1

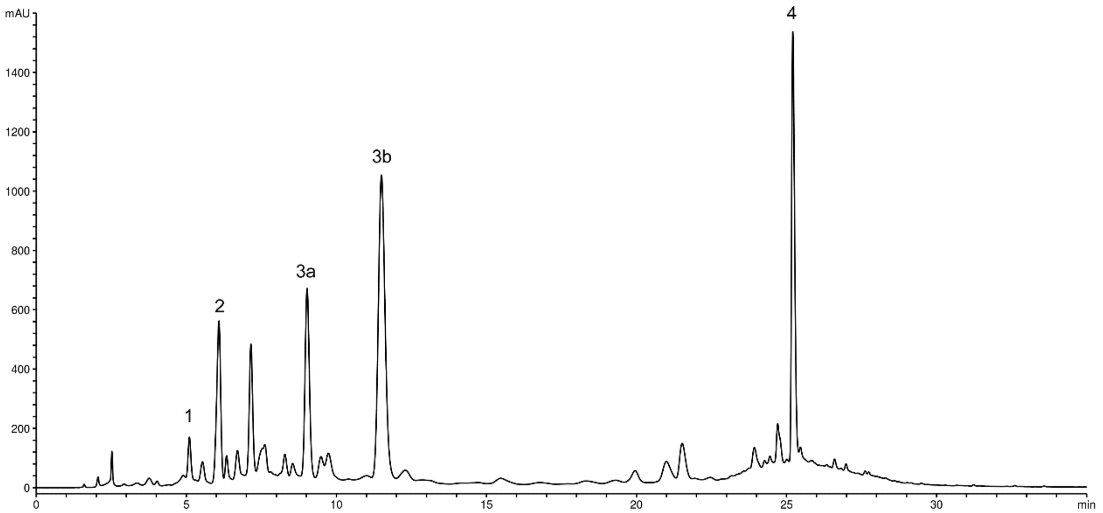

HPLC of PEx. 1. Gallic acid; 2. Punicalin; 3a. Punicalagin-α; 3b. Punicalagin-β; 4. Ellagic acid.

Published by: Čolić et al. Immunomodulatory Properties of Pomegranate Peel Extract in a Model of Human Peripheral Blood Mononuclear Cell Culture. Pharmaceutics 2022, 14, 1140. https://doi.org/10.3390/ pharmaceutics14061140 (With permission of MDPI)
